# Extreme Value Analysis of Wave Heights

**DOI:** 10.6028/jres.099.042

**Published:** 1994

**Authors:** E. Castillo, J. M. Sarabia

**Affiliations:** University of Cantabria Avenida de los Castros s/n, 39005, Santander, Spain

**Keywords:** design, extreme value distributions, wave heights

## Abstract

This paper discusses the most common problems associated with the determination of design wave heights. It analyzes two common methods used in fitting wave data and shows some of the stability or inconsistency problems associated with commonly used distributions. Some methods to obtain confidence intervals, detecting of outliers and treatment of missing data are given.

## 1. Introduction

As in many other fields of engineering, the design of ocean or marine structures is governed by extreme values of wave heights. Several methods have been given in the past for the determination of design values. However, no method is widely accepted by the engineering community.

Traditionally, the analysis of yearly maxima has been considered as a good method for this purpose. However, recently, peak value methods arose as a promising alternative.

The aim of this paper is to compare these two methods and illustrate some of the problems related to their use.

## 2. Two Standard Methods in the Determination of Wave Height Design Values

In this section we analyze the following two well known procedures for obtaining design wave heights: the *peak value method* and the *yearly maxima method.*

The first one employs the peak wave heights of individual storms and thus composes a set of extreme wave data. The second one uses the yearly maxima.

Several authors have criticized the second method in that it discards large wave heights, when they occur in years with large storms, but includes relatively small wave heights which are maxima of calm years.

### 2.1 The Peak Value Method

This method consists of the following steps:

1. Fit the peak values of individual storms to a parametric family of distributions
  $$F_{0}(x;\;\lambda_{0},\;\delta_{0},\;\beta_{0}),$$ (1)
  where *λ*_0_, *δ*_0_ and *β*_0_ are the parameters. In some cases these three parameters can be reduced to two or even to a single one. The fitting of the above family can be done either by using all data or only tail data (Peak over threshold (POT) method).
  It is worthwhile mentioning that this distribution corresponds to the wave height of a storm, that is, we assume that the cdf of the maximum wave height of storms is Eq. (1).
2. Use the following cdf for the maximum wave height in a period of duration *D* years:
  $$F_{0}{\left(x;\;\lambda_{0},\;\delta_{0},\;\beta_{0}\right)}^{Dk},$$ (2)
  where *k* is the mean number of storms per year, or determine the wave height, *x_T_*, associated with a return period *T*, that is, solve, for *x*, the equation
  $$F_{0}{\left(x;\;\lambda_{0},\;\delta_{0},\;\beta_{0}\right)}^{k}=1-\frac{1}{T}.$$ (3)
  Note that the cdf in Eq. (2) implies the assumption of independence of storms.

### 2.2 The Yearly Maxima Method

This method consists of the following steps:

1. Fit the yearly maxima to a parametric family of distributions
  $$F_{1}(x;\;\lambda_{1},\;\delta_{1},\;\beta_{1}),$$ (4)
  where *λ*_1_, *δ*_1_ and *β*_1_ are the new parameters. This is equivalent to assuming that the yearly maxima follow a distribution which belongs to Eq. (4).
2. Use the following formula to extrapolate to the maximum of a period of *D* years:
  $$F_{1}{\left(x;\;\lambda_{1},\;\delta_{1},\;\beta_{1}\right)}^{D},$$ (5)
  or determine the wave height *x* associated with a return period *T*, i.e., solve the equation for *x:*
  $$F_{1}(x;\;\lambda_{1},\;\delta_{1},\;\beta_{1})=1-\frac{1}{T}.$$ (6)

## 3. Some Problems Related to the Data Analysis

In the analysis of data one has to deal with some problems. Among them we mention the following:

- Selection of the families *F*_0_(*x*; *λ*_0_, *δ*_0_, *β*_0_) or *F*_1_(*x*; *λ*_1_, *δ*_1_, *β*_1_)
- Estimation of the parameters of the selected families
- Confidence interval determination
- Outlier detection
- Treatment of incomplete series

### 3.1 Some Distribution Families Used in the Analysis of Wave Data

The most common used distributions in the analysis of wave heights are the following:

1. The Gumbel family
  $$\begin{array}{c} F(x;\;\lambda ,\;\delta )=\exp\left[-\exp\left(\frac{\lambda -x}{\delta }\right)\right];\\ -\;\infty <x<\infty \end{array}$$ (7)
2. The maximal Weibull family
  $$\begin{array}{c} F(x;\;\lambda ,\;\delta ,\;\beta )=\exp\left\{-{\left(\frac{\lambda -x}{\delta }\right)}^{\beta }\right\};\\ x⩽\lambda \end{array}$$ (8)
3. The maximal generalized extreme value or Jenkinson’s family
  $$\begin{array}{c} F(x)=\exp\left\{-{\left[1+\frac{\left(x-B\right)}{kA}\right]}^{-k}\right\};\\ 1+\frac{\left(x-B\right)}{kA}⩾0 \end{array}$$ (9)
4. The minimal Weibull family
  $$\begin{array}{c} F(x;\;\lambda ,\;\delta ,\;\beta )=1-\exp\left\{-{\left(\frac{x-\lambda }{\delta }\right)}^{\beta }\right\};\\ x⩾\lambda \end{array}$$ (10)

The Gumbel, maximal Weibull and maximal Jenkinson’s families are justified from a theoretical point of view, because they are the limit distributions for maxima (see Galambos [5] or Castillo [2]). It is interesting to note that the Jenkinson’s family includes the other two, as particular cases, and the maximal Frechet family (for *k* > 0). The Frechet distribution is not justified in this case because wave heights are physically limited, no matter we deal with shallow or deep waters (see Castillo and Sarabia [3] and [4]).

The minimal Weibull distribution, though widely used, is not theoretically justified in the case of maxima. Its only justification is that its range can be made to be consistent with the positive character of wave heights. In addition, we remind the reader that it belongs to the maximal domain of attraction of the Gumbel type, i.e., it is asymtotically equivalent to a Gumbel distribution of the type Eq. (7).

However, due to the fact that this distribution is widely used in the analysis of wave heights, it seems convenient to make here some comments.

Initially we can say that this distribution has the following advantages:

- For *λ* = 0, its range is (0, ∞), that is, it does not include negative values of the random variable.
- Assuming that the location parameter, due to physical reasons, is fixed to zero, it depends only on two parameters. This makes the estimation process much simpler.
- Its associated domain of attraction is Gumbel type. Thus, it could be used if this were the actual case.

Its main drawbacks are the following:

- Its range is unbounded on the right. This contradicts the physical reality.
- It does not cover the Weibull domain of attraction that could be the real situation.
- It is an asymptotical minimum law.
- It is not stable with respect to maximum operations. Thus, if the minimal Weibull law is satisfied for yearly maxima the maxima of periods of duration different from one year cannot satisfy this law. This problem can be solved by adding an extra parameter to this family, which leads to the extended minimal Weibull family.

Consequently, the minimal Weibull family could be used if and only if we were sure that the domain of attraction of wave heights is of a Gumbel type.

In order to determine the domain of attraction of a given distribution several methods are available, such as the Pickands’ or the curvature methods (see Castillo [2] chapter 6 and Castillo, Galambos and Sarabia [3]).

### 3.2 Estimation Methods

Several methods have been used to estimate the parameters of the families Eqs. (7) to (10). The most important are:

- The maximum likelihood method
- The method of moments
- The least squares method
- The probability paper method
- The Goda’s method
- The percentile method

#### 3.2.1 The Maximum Likelihood Method

This method is based on maximizing the likelihood of data with respect to the parameters. The central idea consists of assuming that the sample comes from a population with parent distribution belonging to a parametric family and choosing the parameter values that maximize the probability of ocurrence of the sample data.

This is the best known method in statistics and it is recognized as the most convenient, due to its statistical properties. It leads to the best estimators, which, in addition, are asymptotically normal. This allows asymptotic confidence intervals for the parameters to be easily obtained. Using the *δ*-method, to be described later, the confidence interval of any regular function of the parameters can be obtained, too. In particular, confidence intervals of percentiles can be obtained in this manner.

In order to estimate an extreme value distribution with the purpose of extrapolation beyond the data range, only high order statistics must be used and the rest must be discarded. Thus, we recommend the method indicated by Castillo [2], in chapter 5.

In the case of the minimal and maximal Weibull families, the estimation process can lead to some problems, either because the likelihood function becomes unbounded (*β* ≤ 1) or because some non-regularities, for some values of the shape parameter (1 < *β* < 2). However, it can be applied to values of the shape parameter larger than or equal to 2 without any problem. Thus, once the estimates are available, it is necessary to check that their values are consistent with the initial hypothesis. Here we give the following recommendations:

- If the shape parameter takes a negative value, this means that the data indicate a Frechet type domain of attraction. This suggests the presence of at least one outlier that gives an erroneous curvature in the right tail.
- If we get a value of *β* ≤ 1, we can think on the presence of outliers. This value of the shapeparameter indicates that the probability density function is increasing in the tail, which contradicts the physical reality.
- If we get 1 < *β* < 2 then the law is far from the Gumbel law (note that Gumbel corresponds to *β* = ∞).
- If the value of the *λ* parameter is less than the maximum of the sample this indicates that there is an outlier.

#### 3.2.2 The Method of Moments

This method consists of equating the moments of the sample to the moments of the theoretical distribution. We use as many moments as there are parameters to be estimated and we get the same number of equations from which the parameters can be obtained. The asymptotic properties of the moment estimates are good but worse than those associated with the maximum likelihood estimates.

This method can also be applied to tail estimation, using the moments of the truncated distribution.

#### 3.2.3 The Least Squares Method

This method consists of minimizing the sum of squares of the differences between the theoretical and the empirical values. There are many versions of this method. In some cases the random variable scale is used to measure the errors and in other cases the probability or the return period scales are used (see chapter 4 of Castillo [2]).

The main advantage of these methods is that they give an explicit solution and do not depend on convergence of any algorithm, as is the case with the maximum likelihood method.

Nevertheless, these methods are sensitive to the plotting position formulas used in the estimation method.

#### 3.2.4 The Probability Paper Method

By probability paper method we understand a visual method, in which the data is drawn on probability paper and a straight line is visually fitted to data.

The main drawback of this method is that it depends on the plotting position formula used in the graphic representation and the subjective criteria for selecting the optimal fit.

#### 3.2.5 The Coda Method

Goda [4] fits a minimal Weibull distribution, truncated at the threshold value *x*_0_, to the right tail of data. By right tail are meant the wave heights above a second threshold value *x*_1_> >*x*_0_.

#### 3.2.6 The Percentile Method

One way of obtaining quick estimates of the parameters of a distribution is by means of the percentile method. This method consists of equating as many percentiles in the sample and the theoretical distribution as the number of parameters to be estimated.

As an illustrative example we use this method for the estimation of the parameters of a three parameter maximal Weibull family.

The cdf of the maximal Weibull distribution is:

$$G(x)=\exp\left[-{\left(\frac{\lambda -x}{\delta }\right)}^{\beta }\right].$$ (11)

Thus, the percentile or order *p* satisfies the equation

$$p=\exp\left[-{\left(\frac{\lambda -x_{p}}{\delta }\right)}^{\beta }\right],$$ (12)

from which we get

$$x_{p}=\lambda -\delta {\left(-\log\;p\right)}^{1/\beta }.$$ (13)

Equating the three percentiles of orders *p*_1_, *p*_2_, *p*_3_ of sample and population, we get the following system of equations:

$$x_{p_{i}}=\lambda -\delta {\left(-\log\;p_{i}\right)}^{1/\beta };\;i=1,\;2,\;3,$$ (14)

where *p*_i_ can be written, using the Gringorten’s formula, as:

$$p_{i}=\frac{i-0.44}{n+0.12},$$ (15)

where *i* is the rank of the order statistic associated with *p_i_.*

From Eq. (14) we get

$$\frac{x_{p_{2}}-x_{p_{1}}}{x_{p_{3}}-x_{p_{2}}}=\frac{{\left(-\log\;p_{2}\right)}^{1/\beta }-{\left(-\log\;p_{1}\right)}^{1/\beta }}{{\left(-\log\;p_{3}\right)}^{1/\beta }-{\left(-\log\;p_{2}\right)}^{1/\beta }},$$ (16)

which depends only on the parameter *β* and thus, it can be easily solved by an iterative method, as the bisection method for example, with a personal computer. Once *β* is known, the values of *λ* and *δ* can be obtained from any two of the equations in Eq. (14). For example:

$$\begin{array}{c} \delta =\frac{x_{p_{2}}-x_{p_{1}}}{{\left(-\log\;p_{1}\right)}^{1/\beta }-{\left(-\log\;p_{2}\right)}^{1/\beta }};\\ \lambda =x_{p_{i}}+\delta {\left(-\log\;p_{1}\right)}^{1/\beta } \end{array}$$ (17)

For the estimates to be consistent with the model we must have

$$\lambda >\max(x_{1},\;x_{2},\ldots ,x_{n})$$ (18)

where *(x*_1_, *x*_2_, …,*x_n_*) is the sample.

If the percentiles are arbitrarily chosen, this inconsistency can easily appear. Thus, it is good practice to choose as one of the percentiles the maximum of the sample *x*_(_*_n_*_)_.

In addition, if we are dealing with a tail estimation we must choose the adequate percentiles, that is, percentiles in it.

In order to improve the quality of the estimates we can use three groups of percentiles instead of three percentiles, that is, replace the system Eq. (14) by the system

$$\begin{array}{c} \frac{1}{m_{j}}\sum_{i=k_{j}}^{i=k_{j}+m_{j}-1}x_{p_{i}}=\lambda -\frac{\delta }{m_{j}}\sum_{i=k_{j}}^{i=k_{j}+m_{j}-1}{\left(-\log\;p_{i}\right)}^{1/\beta };\\ j=1,\;2,\;3 \end{array}$$ (19)

where *m_j_*, (*j* = 1, 2, 3) are the numbers of percentiles included in each group. With this, equation Eq. (16) becomes Eq. (20)

$$\begin{array}{c} \frac{\frac{1}{m_{2}}\sum_{i=k_{2}}^{i=k_{2}+m_{2}-1}x_{p_{i}}-\frac{1}{m_{1}}\sum_{i=k_{1}}^{i=k_{1}+m_{1}-1}x_{p_{i}}}{\frac{1}{m_{3}}\sum_{i=k_{3}}^{i=k_{3}+m_{3}-1}x_{p_{i}}-\frac{1}{m_{2}}\sum_{i=k_{2}}^{i=k_{2}+m_{2}-1}x_{p_{i}}}=\\ \frac{\frac{1}{m_{2}}\sum_{i=k_{2}}^{i=k_{2}+m_{2}-1}{\left(-\log\;p_{i}\right)}^{1/\beta }-\frac{1}{m_{1}}\sum_{i=k_{1}}^{i=k_{1}+m_{1}-1}{\left(-\log\;p_{i}\right)}^{1/\beta }}{\frac{1}{m_{3}}\sum_{i=k_{3}}^{i=k_{3}+m_{3}-1}{\left(-\log\;p_{i}\right)}^{1/\beta }-\frac{1}{m_{2}}\sum_{i=k_{2}}^{i=k_{2}+m_{2}-1}{\left(-\log\;p_{i}\right)}^{1/\beta }}. \end{array}$$ (20)

#### 3.2.7 Plotting Position Formulas

There is much controversy about the plotting position formulas to be used for representing data on probability paper and the posterior estimation by least squares methods.

The resulting estimates are sensitive to the plotting position formulas being used. This confirms the fact that the least squares method is not optimal. Note that maximum likelihood or moment methods do not depend on plotting positions.

The discussion of the appropriateness of various formulas is intended to avoid or reduce some of the errors involved (in this case authors recommend using formulas leading to unbiased estimators).

However, we mention here that all plotting position formulas are asymptotically equivalent.

### 3.3 *δ*-Method

The *δ*-method (Bishop, Fienberg, and Holland [1]) allows us to obtain confidence intervals of certain regular functions of the parameters, as functions of the parameter estimates, and its variance-covariance matrix.

Let

$$\eta_{i}=h_{i}(\lambda_{1},\;\lambda_{2},\ldots ,\lambda_{s});\;i=1,\;2,\ldots ,k$$ (21)

be *k* functions of the set of parameters *λ*_1_, *λ*_2_, …, *λ*_s_,. Then, according to the *δ*-method,

$$\begin{array}{c} ({\hat{\eta }}_{1},\;{\hat{\eta }}_{1},\ldots ,{\hat{\eta }}_{k})=(h_{1}({\hat{\lambda }}_{1},\;{\hat{\lambda }}_{2},\ldots ,{\hat{\lambda }}_{s}),\\ h_{2}({\hat{\lambda }}_{1},\;{\hat{\lambda }}_{2},\ldots ,{\hat{\lambda }}_{s}),\ldots ,h_{k}({\hat{\lambda }}_{1},\;{\hat{\lambda }}_{2},\ldots ,{\hat{\lambda }}_{s})) \end{array}$$ (22)

is an estimator of (*η*_1_, *η*_2_, …, *η_k_*) which is asymptotically normal and has mean

$$\begin{array}{c} (h_{1}(\lambda_{1},\;\lambda_{2},\ldots ,\;\lambda_{s}),\;h_{2}(\lambda_{1},\;\lambda_{2},\ldots ,\;\lambda_{s}),\ldots ,\\ h_{k}(\lambda_{1},\;\lambda_{2},\ldots ,\lambda_{s})) \end{array}$$ (23)

and variance-covariance matrix

$$\begin{array}{c} \sum^{*}=\left[\begin{array}{cccc} \frac{\partial h_{1}}{\partial \lambda_{1}} & \frac{\partial h_{2}}{\partial \lambda_{1}} & \ldots & \frac{\partial h_{k}}{\partial \lambda_{1}}\\ \frac{\partial h_{1}}{\partial \lambda_{2}} & \frac{\partial h_{2}}{\partial \lambda_{2}} & \ldots & \frac{\partial h_{k}}{\partial \lambda_{2}}\\ \ldots & \ldots & \ldots & \ldots \\ \frac{\partial h_{1}}{\partial \lambda_{s}} & \frac{\partial h_{2}}{\partial \lambda_{s}} & \ldots & \frac{\partial h_{k}}{\partial \lambda_{s}} \end{array}\right]\\ \;\sum \left[\begin{array}{cccc} \frac{\partial h_{1}}{\partial \lambda_{1}} & \frac{\partial h_{2}}{\partial \lambda_{1}} & \ldots & \frac{\partial h_{k}}{\partial \lambda_{1}}\\ \frac{\partial h_{1}}{\partial \lambda_{2}} & \frac{\partial h_{2}}{\partial \lambda_{2}} & \ldots & \frac{\partial h_{k}}{\partial \lambda_{2}}\\ \ldots & \ldots & \ldots & \ldots \\ \frac{\partial h_{1}}{\partial \lambda_{s}} & \frac{\partial h_{2}}{\partial \lambda_{s}} & \ldots & \frac{\partial h_{k}}{\partial \lambda_{s}} \end{array}\right] \end{array}$$ (24)

where *Σ* is the variance-covariance matrix of $\left({\hat{\lambda }}_{1},{\hat{\lambda }}_{2},\ldots ,{\hat{\lambda }}_{s}\right)$.

### 3.4 Estimation of Percentiles of the Maximal Weibull Distribution

As a simple example of the *δ*-method we give the confidence interval of one percentile of the three parameter maximal Weibull distribution. We assume that the parameter *β* is larger than 2.

#### 3.4.1 Point Estimate

The percentile *x_p_* of the maximal Weibull distribution is;

$$x_{p}=\lambda -\delta {\left(-\log\;p\right)}^{1/\beta }.$$ (25)

Thus, according to the invariance principle, the maximum likelihood estimator of that percentile, is:

$${\hat{x}}_{p}=\hat{\lambda }-\hat{\delta }{\left(-\log\;p\right)}^{1/\beta }$$ (26)

#### 3.4.2 Maximum Likelihood Estimators: Asymptotic Theory

We assume here that the sample consists of those observed values above the threshold value *t* (type II censoring), that is, the probability density function is given by

$$f_{t}(x)=\frac{f(x;\lambda ,\delta ,\beta )}{1-F(t;\lambda ,\delta ,\beta )}$$ (27)

and *f* and *F* are the pdf and cdf of the maximal Weibull family.

The maximal Weibull distribution satisfies the necessary regularity conditions for the asymptotic normality if *β* ≥ 2. Thus, if we have a sufficiently large sample coming from a population with maximal Weibull parent, we can write:

$$\sqrt{n}((\hat{\lambda },\hat{\delta },\hat{\beta })-(\lambda ,\;\delta ,\beta ))\overset{D}{\to }N(\underset{\to }{0},\;\Sigma )$$ (28)

where

$$\Sigma ={\left(I+J\right)}^{-1},$$ (29)

where *I* = (*a_ij_*) is the information matrix associated with the maximal Weibull family, that is,

$$a_{11}=I_{\lambda \lambda }=\frac{{\left(\beta -1\right)}^{2}}{\delta^{2}}\Gamma (1-\frac{2}{\beta })$$ (30)

$$a_{12}=I_{\lambda \delta }=-\frac{\beta^{2}}{\delta^{2}}\Gamma (2-\frac{1}{\beta })$$ (31)

$$a_{13}=I_{\lambda \beta }=\frac{-1}{\beta }\Gamma (1-\frac{1}{\beta })+\frac{1}{\delta }\Gamma (2-\frac{1}{\beta })+\frac{1}{\delta }\Gamma (2-\frac{1}{\beta })$$ (32)

$$a_{22}=I_{\delta \delta }=\frac{\beta^{2}}{\delta^{2}}$$ (33)

$$a_{23}=I_{\delta \beta }=-\frac{1}{\delta }\Gamma \prime (2)$$ (34)

$$a_{33}=I_{\beta \beta }=\frac{1}{\beta^{2}}(1+\Gamma^{\prime \prime }(2))$$ (35)

and *J* is the matrix of the second order partial derivatives, with respect to the parameters of the model, of the function

G(t;λ,δ,β)=log[1−F(t;λ,δ,β)].

If we consider now the function:

$$f(\lambda ,\delta ,\beta )=x_{p}=\lambda -\delta {\left(-\log\;p\right)}^{1/\beta },$$ (36)

with partial derivatives:

$$\begin{array}{l} f_{1}=\frac{\partial f}{\partial \lambda }=1\\ f_{2}=\frac{\partial f}{\partial \delta }=-\;{\left(-\log\;p\right)}^{1/\beta }\\ f_{3}=\frac{\partial f}{\partial \beta }=\frac{\delta }{\beta^{2}}\log\;((-\log\;p)){\left(-\log\;p\right)}^{1/\beta } \end{array}$$ (37)

then, by the *δ*-method we have:

$$\sqrt{n}(f(\hat{\lambda },\hat{\delta },\hat{\beta })-f(\lambda ,\delta ,\beta ))\overset{\upsilon }{\to }N(\underline{0},\;\Sigma^{*})$$ (38)

where

$$\Sigma^{*}=(f_{1},f_{2},f_{3})\Sigma (f_{1},f_{2},f_{3}{)}^{\prime },$$ (39)

from which the confidence interval for the percentile *x_p_* at level *α* becomes:

$$({\hat{x}}_{p}-z_{1-a/2}\frac{\Sigma^{*1/2}}{\sqrt{n}};{\hat{x}}_{p}+z_{1-a/2}\frac{\Sigma^{*1/2}}{\sqrt{n}}).$$ (40)

### 3.5 Outlier Detection

In this section we give a method to detect the presence of outliers in the sample data. The method is based on the fact that if we make the following change of variable:

Y=F(X) (41)

where *F*(*x*) is the cdf of *X*, the resulting random variable, *Y*, is uniform *U*(0,1).

In addition we know that the maximum of a random sample of size *n* coming from a standard uniform parent has cdf

$$F_{Y_{\max}}(y)=y^{n}=\text{Prob}[Y_{\max}⩽y].$$ (42)

We shall say that the sample maximum is one outlier if the probability of being exceeded is very small. Thus, the value *y*_0_ can be considered as critical for the maximum value of the sample if

$$\text{Prob}[Y_{\max}>y_{0}]=F_{Y_{\max}}(y_{0})=1-y_{\upsilon }^{\eta }=\alpha$$ (43)

with *a* very small (0.01, 0.05, etc.). Then, we get

$$y_{0}={\left(1-\alpha \right)}^{t/n}$$ (44)

This critical value refers to the random variable *Y.* Thus, we need to obtain *X* by means of the inverse of Eq. (41). As one example, for the maximal Weibull distribution we get

$$x_{0}=\hat{\lambda }-\hat{\delta }{\left[-\log{\left(1-\alpha \right)}^{t/n}\right]}^{1/\beta }.$$ (45)

### 3.6 Treatment of Incomplete Series

If we know about the existence of *r* storms in a given series, but we ignore the peak intensities we can perform an estimate based on the known peaks and then make a correction for the unknown peaks. This means estimating the cdf with the known peaks and raise to the power (*n+r*)/*n*, where *n* and *r* are the number of known and unknown peaks, respectively.

## 4. Critical Analysis

In this section we analyze the previous methods and discuss some of their inconsistencies.

### 4.1 Inconsistencies due to the Lack of Stability With Respect to Maximum Operations

When several design methods are recognized by the engineering community a certain consistency in the respective results should be expected. We shall see that this is not the case for some of the previous methods.

Let us assume that we try to fit the minimal Weibull family

$$F(x;\lambda_{0},\delta_{0},\beta_{0})=1-\exp\left\{-{\left(\frac{x-\lambda_{0}}{\delta_{0}}\right)}^{\beta_{0}}\right\},$$ (46)

where *λ*_0_, *δ*_0_ and *β*_0_ are the parameters. Then, Eq. (2) transforms to

$$\begin{array}{c} F_{D}(x;\lambda_{0},\delta_{0},\beta_{0})=F{\left(x;\lambda_{0},\delta_{0},\beta_{0}\right)}^{Dk}=\\ {\left\{1-\exp\left[-{\left(\frac{x-\lambda_{0}}{\delta_{0}}\right)}^{\beta_{0}}\right]\right\}}^{Dk} \end{array}$$ (47)

and the wave height associated with a return period *T* becomes Eq. (3):

$$x_{T}=\lambda_{0}+\delta_{0}{\left\{-\log\left[1-{\left(1-\frac{1}{T}\right)}^{1/k}\right]\right\}}^{1/\beta o}$$ (48)

Let us assume that now we also use the minimal Weibull family in Eq. (4):

$$F_{1}(x;\lambda_{1},\delta_{1},\beta_{1})=1-\exp\left\{-{\left(\frac{x-\lambda_{1}}{\delta_{1}}\right)}^{\beta_{1}}\right\},$$ (49)

where *λ*_t_, *δ*_t_ and *β*_1_ are the new parameters. Then, Eq. (5) becomes

$$\begin{array}{c} F_{D}(x;\lambda_{1},\delta_{1},\beta_{1})=F_{1}{\left(x;\lambda_{1},\delta_{1},\beta_{1}\right)}^{D}=\\ {\left\{1-\exp\left[-{\left(\frac{x-\lambda_{1}}{\delta_{1}}\right)}^{\beta_{1}}\right]\right\}}^{D} \end{array}$$ (50)

and the wave height associated with a return period *T*, from Eq. (6), is

$$x_{T}=\lambda_{1}+\delta_{1}{\left\{-\log\left[1-\left(1-\frac{1}{T}\right)\right]\right\}}^{1/\beta_{1}}$$ (51)

The minimal Weibull model is inconsistent in the following sense: It is not stable with respect to maximum operations, that is, when the cdf is raised to a given power *s* ≠ 1, then, the resulting cdf is not minimal Weibull. Thus, though Eq. (46) is minimal Weibull, Eq, (47) is not minimal Weibull for *Dk* ≠ 1. In other words, if we assume a minimal Weibull distribution for the peaks of storms, the yearly maxima cannot be minimal Weibull and vice versa. In fact for equations Eqs. (47) and (48) to be identical to Eqs. (50) and (51), respectively, i.e., for consistency, we must have

$$\lambda_{0}=\lambda_{1},\delta_{0}=\delta_{1},\beta_{0}=\beta_{1},k=1$$ (52)

which implies *k* = 1, that is a mean number of one storm per year, which is not the case.

However, if, instead of using the minimal Weibull family we use the maximal Gumbel family

$$F_{0}(x;\lambda_{0},\delta_{0})=\exp\left[-\exp\left(\frac{\lambda_{0}-x}{\delta_{0}}\right)\right],$$ (53)

then, Eqs. (47), (48), (50) and (51) become

$$F_{D}(x;\lambda_{0},\delta_{0})={\left\{\exp\left[-\exp\left(\frac{\lambda_{0}-x}{\delta_{0}}\right)\right]\right\}}^{Dk}$$ (54)

$$x_{T}=\lambda_{0}-\delta_{0}\log\left[-\log{\left(1-\frac{1}{T}\right)}^{1/k}\right]$$ (55)

$$F_{D}(x;\lambda_{1},\delta_{1})={\left\{\exp\left[-\exp\left(\frac{\lambda_{1}-x}{\delta_{1}}\right)\right]\right\}}^{D}$$ (56)

and

$$x_{T}=\lambda_{1}-\delta_{1}\;\log\left[-\log\left(1-\frac{1}{T}\right)\right]$$ (57)

and, taking into account that

$$\begin{array}{l} {\left\{\exp\left[-\exp\left(\frac{\lambda_{1}-x}{\delta_{1}}\right)\right]\right\}}^{D}=\\ {\left\{\exp\left[-\exp\left(\frac{\lambda_{1}-x}{\delta_{1}}\right)\right]\right\}}^{Dk\;1/k}=\\ {\left\{\exp\left[-\exp\left(\frac{\lambda_{1}-\delta_{1}\log k-x}{\delta_{1}}\right)\right]\right\}}^{Dk} \end{array}$$ (58)

the coincidence of the pairs Eqs. (54)–(56) and Eqs. (55)–(57) implies

$$\delta_{0}=\delta_{1}\;y\lambda_{0}=\lambda_{1}-\delta_{1}\log k.$$ (59)

That is, the coincidence of both is possible for any value of *k.*

The same conclusion is valid for any of the Weibull Eq. (8) or the Jenkinson’s Eq. (9) families.

### 4.2 Inconsistencies Associated With the Lack of Stability With Respect to Truncation

Goda’s method is inconsistent for the following reasons:

1. It gives different estimators for different values of *x*_0_.
2. If the truncated distribution belongs to the minimal Weibull family it cannot belong for a different threshold value. Thus, different designers using different threshold values necessarily arrive to different models.

In the following paragraphs we shall make a detailed analysis of this problem.

With respect to the first inconsistency it is clear that because the method only uses the data above the second threshold value *x*_1_, the resulting estimates should be independent on the First threshold value *x*_0_

In relation to the second inconsistency, the model should be stable with respect to truncations. With the purpose of clarifying this idea, let us assume that we choose a family of candidate distributions *H*(*x*;*y*), where the second argument *y* is one parameter, which, without loss of generality, can be assumed to be the threshold value. Then, if the wave height exceeding *z* has as cdf the function *H*(*x*,*z*), then, the wave height exceeding *y* should have a cdf given by

$$\frac{H(x;z)-H(y;z)}{1-H(y;z)}=H(x;y),$$ (60)

where the right hand term arises from the consistency condition that expresses that the family *H*(*x;z*) remains valid for any value of the threshold parameter, which in this case is *y*.

Equation (60) is a functional equation. Its general solution can easily be obtained by making *z* = *z*_0_, that is,

$$H(x;y)=\frac{G(x)-G(y)}{1-G(y)},$$ (61)

where

$$G(x)=H(x;z_{0}).$$ (62)

For *H*(*x;y*) to be a cdf, then *G*(*x*) must also be a cdf.

Equation (61) proves that any consistent family *H*(*x;y*) must come from another family *G*(*x*) by means of a truncation procedure.

The minimal Weibull family, used by Goda, does not satisfy this condition. Thus, it is inconsistent.

With the purpose of having a consistent family in the two previously given senses, one solution would consist of assuming *G*(*x*) to be extended minimal Weibull with null location parameter. This would imply that the sample data above the threshold value *x*_1_ should be fitted to the family

$$H(x;y,\delta ,\beta )=\frac{{\left[F(x;\delta ,\beta )\right]}^{n}-{\left[F(y;\delta ,\beta )\right]}^{n}}{1-{\left[F(y;\delta ,\beta )\right]}^{n}},$$ (63)

where

$$F(x;\delta ,\beta )=1-\exp\left[-{\left(\frac{x}{\delta }\right)}^{\beta }\right],$$ (64)

Nevertheless, we remind the reader that this solution can be satisfactory only in the case of aparent distribution in the domain of attraction for maxima of a Gumbel type.

Consequently, as a summary, we recommend to fit the sample data above the threshold to one of the following three families:

- If the domain of attraction is Weibull type, fit the right tail to the maximal Weibull family
  $$F_{0}(x;\lambda_{0},\delta_{0},\beta_{0})=\exp\left\{-{\left(\frac{\lambda_{0}-x}{\delta_{0}}\right)}^{\beta_{0}}\right\}$$ (65)
- if the maximal domain of attraction is Gumbel type, fit the right tail to the maximal Gumbel family
  $$F_{0}(x;\lambda_{0},\delta_{0},)=\exp\left[-\exp\left(\frac{\lambda_{0}-x}{\delta_{0}}\right)\right]$$ (66)
  or to the extended minimal Weibull family
  $${\begin{array}{c} F_{0}(x;\lambda ,\delta ,\beta )=\left\{1-\exp\left[-{\left(\frac{x}{\delta }\right)}^{\beta }\right]\right\}\\ x⩾0 \end{array}}^{\eta };$$ (67)
  where *β*, *δ* and *η* are the parameters to be estimated. In the last case we are assuming that the cdf of the maximum wave height in an indeterminate period, to be estimated, is minimal Weibull.

Note that fitting the right tail means fitting a truncated model with basic distribution given by Eqs. (65), (66) or (67).

All these models are consistent in the previously mentioned sense.

### 4.3 Inconsistencies Associated With the Use of *H_s_* and *T*_z_

It is very common in the Ocean Engineering field to work with the significant wave height, *H_s_*, and period, *T_z_*, as the basic variables for extreme value analysis of waves. However this is not correct because *T_z_* is the mean zero up-crossing period and *H_s_* is defined as the mean of the 1/3 largest waves. These two random variables are convenient to justify normality assumptions in wave spectra, but cannot be accepted if an extreme value analysis of wave height, *H*, is to be performed. In fact, distributions in different domain of attraction types can lead to the same distribution for *H_s_* and/or *T_z_*, thus, obscuring the tail properties of single waves.

## 5. Conclusions

From all the above we get the following conclusions:

1. The most convenient families to fit wave height data in the tails are:
  - The maximal Weibull family
  - The maximal Gumbel family
    However, the extended minimal Weibull family can be used too.
    Before fitting the Gumbel or the extended minimal Weibull families, the domain of attraction for maxima must be checked using, for example, the Pickands or the curvature methods. For the estimation of the parameters, the maximum likelihood or the method of moments applied to the truncated distributions is recommended.
    In the case of the maximal Weibull family, the shape parameter *β* must be larger than unity. If it is not, the data suggests an increasing probability density function in the tail, which contra-dicts the reality.
2. It is recommended the elimination of outliers by means of the following iterative method:
  - Estimate all parameters with all data but the maximum
  - Check for the outlier character of the maximum by the previously indicate method
  - If it is an outlier, remove the maximum and start the process again; if it is not, repeat the estimation with all the valid data
3. If there are missing data correct the obtained cdf by raising to the power (*n* + *r*)/*n* where *n* and *r* are the number of known and unknown data, respectively.
4. Significant wave height *H_s_* and mean up-crossing periods *T_z_* are not adequate variables to analyze the extreme value behaviour of wave heights.
